# Exploration of factors affecting hemodynamic stability following pheochromocytoma resection - cohort study

**DOI:** 10.3389/fendo.2024.1336128

**Published:** 2024-04-08

**Authors:** Lidan Liu, Lihua Shang, Yimeng Zhuang, Xiaojing Su, Xue Li, Yumeng Sun, Bo Long

**Affiliations:** Department of Anesthesiology, Shengjing Hospital of China Medical University, Shenyang, China

**Keywords:** haemodynamic instability, pheochromocytoma, postoperative hypotension, vasoactive drug, perioperative management

## Abstract

**Purpose:**

Surgery is the only way to cure pheochromocytoma; however, postoperative hemodynamic instability is one of the main causes of serious complications and even death. This study’s findings provide some guidance for improved clinical management.

**Patients and methods:**

This study was to investigate the factors leading to postoperative hemodynamic instability in the postoperative pathology indicated pheochromocytoma from May 2016 to May 2022. They were divided into two groups according to whether vasoactive drugs were used for a median number of days or more postoperatively. The factors affecting the postoperative hemodynamics in the perioperative period (preoperative, intraoperative, and postoperative) were then evaluated.

**Results:**

The median number of days requiring vasoactive drug support postoperatively was three in 234 patients, while 118 (50.4%) patients required vasoactive drug support for three days or more postoperatively. The results of the multivariate analysis indicated more preoperative colloid use (odds ratio [OR]=1.834, confidence interval [CI]:1.265–2.659, P=0.001), intraoperative use of vasoactive drug (OR=4.174, CI:1.882–9.258, P<0.001), and more postoperative crystalloid solution input per unit of body weight per day (ml/kg/d) (OR=1.087, CI:1.062–1.112, P<0.001) were risk factors for predicting postoperative hemodynamic instability. The optimal cutoff point of postoperative crystalloid use were 42.37 ml/kg/d.

**Conclusion:**

Hemodynamic instability is a key issue for consideration in the perioperative period of pheochromocytoma. The amount of preoperative colloid use, the need for intraoperative vasoactive drugs, and postoperative crystalloid solution are risk factors for predicting postoperative hemodynamic instability (registration number: ChiCT2300071166).

## Introduction

Pheochromocytomas are endocrine tumors of adrenal medullary origin, characterized by the secretion of catecholamines, including norepinephrine, epinephrine, and dopamine. Subsequently, severe cardiovascular disease is induced, including severe hypertensive crisis, arrhythmias, myocardial infarction, and acute heart failure (1, 2). While surgical resection is the only cure for this disease, the presence of hemodynamic fluctuations in the perioperative period seriously endangers the patient’s life. For example, tracheal intubation, change of position, the establishment of pneumoperitoneum, and touching the tumor will cause massive secretion of catecholamines and a dramatic increase in blood pressure, while a sudden decrease in catecholamines following tumor removal will result in persistent intractable hypotension and hypoglycemia (3).

The predictors of intraoperative hemodynamic instability in pheochromocytoma have been reported to include preoperative blood pressure control level, tumor size, preoperative catecholamine level, and surgical approach (4, 5), while the predictors of postoperative hypotension include preoperative beta-blocker use (6), type of preoperative alpha-blocker use (7), catecholamine secretion level (7), and tumor size (8). In fact, various studies have reported that tumor size is not an influencing factor in predicting hemodynamic instability; however, this was likely because the size of the tumor in these studies was comparatively small (7). Meanwhile, studies have also reported no difference in the effect of different surgical approaches (9, 10).

Most current studies focus on preoperative and intraoperative management, with few studies focusing on postoperative management. Both the hemodynamic effects of large intraoperative catecholamine release and the dramatic decrease in catecholamine levels following tumor resection may persist into the postoperative period, and the investigation of postoperative management should not be neglected in view of reducing hemodynamic fluctuations. The aim of this study was to explore the factors affecting the use of vasoactive drugs in the postoperative period.

## Materials and methods

### Study design and patient selection

**Figure 1:**
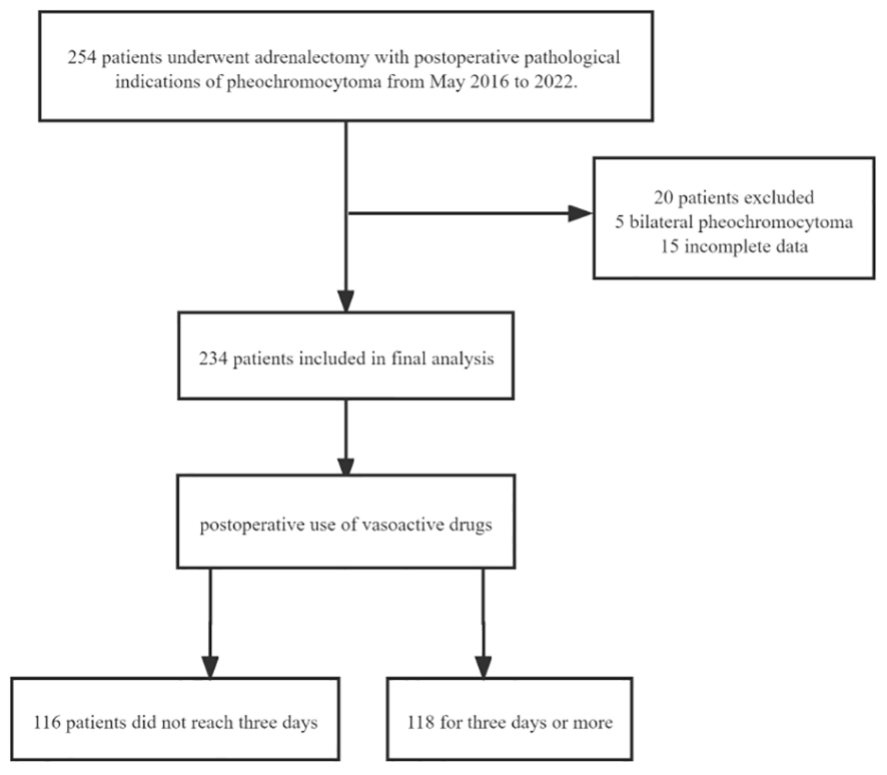
Flow diagram of the study.

The study has been reported in line with the STROCSS criteria (11) and ethical approval was granted, which applied for a waiver of informed consent. The study has been registered in the Chinese Clinical Trial Registry. This was a single-center retrospective study conducted between May 2016 and 2022. A total of 254 adrenalectomy patients from the same surgical group of operators with postoperative pathology of pheochromocytoma were included.20 were excluded. Among them, five were excluded due to bilateral pheochromocytoma, and fifteen were excluded due to incomplete data. The final 234 patients were included in the statistical analysis and were divided into two groups according to the median time(whether it is≥3 days) of the postoperative use of vasoactive drugs (**Figure 1**).

### Preoperative management

Preoperative treatment with terazosin or other antihypertensive agents is required for patients with biochemical parameters, clinical symptoms, or imaging suspicion of pheochromocytoma. The need for beta-adrenergic receptor blockers to control the heart rate is determined by the presence of tachycardia. For patients with severe preoperative clinical symptoms and a large tumor size, as determined via imaging, appropriate volume expansion therapy (crystalloid/colloid/albumin/plasma/erythrocytes) should be administered 2–3 days prior to surgery. Here, the preoperative control criteria were orthostatic hypotension of <130/80 mm Hg and a heart rate of <90/min.

### Outcomes

The basic preoperative information (gender, age, weight, body mass index [BMI]), the presence of diabetes, coronary heart disease comorbidity, American Society of Anesthesiologists classification, tumor size, preoperative blood pressure, preoperative medication, preoperative ionic disturbances, preoperative fluid replacement, blood transfusion and albumin transfusion were recorded. The mode of surgery, the duration of surgery, whether intraoperative vasoactive drugs were used, the amount of norepinephrine used, intraoperative blood and fluid transfusion, intraoperative urine volume, the amount of tachyphylaxis used were also recorded. In addition, whether the patients were admitted to the intensive care unit following surgery, as well as information related to postoperative blood transfusion, albumin transfusion, amount of rehydration fluid, postoperative 24 h urine volume, tachyphylaxis use, and postoperative hospitalization time were examined.

### Statistical analysis

The statistical analysis was conducted using IBM SPSS Statistics for Windows, Version 25.0. The normality of the continuous variables was determined using the Shapiro–Wilk test, with the variables following normal distribution expressed as mean ± standard deviation (SD). The non-normally distributed continuous variables were presented in terms of median (interquartile range). The means of two continuous normally distributed variables were compared using the independent samples Student’s t-test, while the Mann–Whitney U-test was used to compare two continuous non-normally distributed variables. The categorical variables were presented in terms of quantity (percentage), with a chi-square test and Fisher’s exact test used to compare these variables.

To ascertain the association between duration of use and the preoperative, intraoperative, and postoperative factors, univariate analysis was performed in terms of patients who used norepinephrine for a number of days at or above the median and those who did not. Factors with a P-value of <0.1 in the univariate analysis were included in a multivariate binary logistic regression model. Variance inflation factors (VIFs) were used to evaluate the collinearity, and any variables with severe collinearity (VIF≥10) were excluded from the multivariate analysis. Multivariate logistic regression analysis was performed to determine the predictors of postoperative norepinephrine use reaching and exceeding the median number of days, with the variables selected using a forward approach. The cutoff values and the area under the curve values for the continuous variables that were independent risk factors for norepinephrine use reaching and exceeding the median number of days were calculated using receiver operating characteristic curve analysis. A P-value of <0.05 was considered to be statistically significant.

## Results

Among the included 234 patients, the median time to postoperative need for norepinephrine for hypotension was three days, and compared with the patients who did not reach three days of vasoactive drug use, those who reached or exceeded this point had a lower BMI (23.1 vs. 24.2 kg/m2, P=0.012), a larger tumor (5.0 vs. 4.4 cm, P=0.003), lowerer doses preoperative doxazosin use (4.5 vs. 5 mg, P=0.001), more preoperative crystalloid solution (1225 vs. 0 ml, P<0.001), and higher colloid use (500 vs. 0 ml, P<0.001). There was no significant difference in age and sex between the two groups. The detailed intraoperative information is provided in **Table 1**.

**Table 1:** Demographics and perioperative data between two groups.

Variables	Vasopressor use <3d N=116	Vasopressor use ≥3d N=118	Total N=234	P-value
Female	58 (50.0%)	61 (51.7%)	119 (50.9%)	0.795
Age (years)	54.5 (45.3-62.0)	53.0 (45.8-62.0)	52.5 (45.8-62.0)	0.715
Body weight (kg)	67.1 ± 10.3	62.5 ± 11.0	64.8 ± 10.9	0.645
BMI (kg/m2)	24.2 (21.8-25.4)	23.1 (20.8-24.6)	23.6 (21.4-24.8)	0.012
Diabetes mellitus	32 (27.6%)	38 (32.2%)	70 (29.9%)	0.441
Coronary artery disease	15 (12.9%)	12 (10.2%)	27 (11.5%)	0.509
Hypertension	49 (42.2%)	50 (42.4%)	99 (42.3%)	0.984
Maximal size (cm)	4.4 (3.0-6.0)	5.0 (4.0-6.8)	4.9 (3.5-6.0)	0.003
ASA				<0.001
II	81 (69.8%)	75 (63.6%)	156 (66.7%)	
III	35 (30.2%)	43 (36.4%)	78 (33.3%)	
terazosin (mg)	5 (0-8)	4.5 (0-22)	2 (0-16)	0.001
other antihypertensive agents	29 (25.0%)	31 (26.3%)	60 (25.6%)	0.824
low potassium	9 (7.8%)	7 (5.9%)	16 (6.8%)	0.580
crystal (ml)	0 (0-1500)	1225 (0-2500)	1000 (0-2000)	<0.001
colloid (ml)	0 (0-500)	500 (0-1500)	250 (0-1000)	<0.001
RBC (U)	0 (0-0)	0 (0-0)	0 (0-0)	0.199
Plasma (ml)	0 (0-0)	0 (0-0)	0 (0-0)	0.010
albumin (g)	0 (0-0)	0 (0-10)	0 (0-0)	<0.001

Continuous variables with normal distribution were reported as the mean ± standard deviation (SD); non-normal continuous variables were expressed as median (interquartile range); categorical variables were reported as number(percentage).

BMI, body mass index; ASA, American Society of Anesthesiologists.

Compared with the patients who did not reach the median number of days of postoperative vasopressor use, a higher proportion of patients who received intraoperative vasopressor treatment required vasoactive drug support to meet or exceed the median number of days after surgery (85.6% vs. 46.6%, P<0.001), as well as higher intraoperative norepinephrine use (0.6 vs. 0 mg, P<0.001) and more intraoperative fluid infusions (3000 vs. 2,500 ml, P=0.001). The detailed intraoperative information is provided in **Table 2**. The patients who reached or exceeded the median number of days of postoperative crystalloid solution input (57.4 vs. 26.2 ml/kg/d, P<0.001) had a longer postoperative hospital stay compared with those who did not reach the median number of days of postoperative booster use (ten vs. seven days P<0.001). Detailed information is provided in **Table 3**.

**Table 2:** Intraoperative data of patients between two groups.

Variables	Vasopressor use <3d N=116	Vasopressor use ≥3d N=118	Total N=234	P-value
Laparoscopy	101 (87.0%)	103 (87.3%)	204 (87.2%)	0.960
Intraoperative vasopressor support	54 (46.6%)	101 (85.6%)	155 (66.2%)	<0.001
Duration of surgery (minutes)	150 (120-180)	169 (120-210)	150 (120-205.2)	0.072
Total infusion volume (ml)	2500 (1725-3300)	3000 (2200-3800)	2600 (2000-3500)	0.001
Crystal (ml)	1775 (1300-2500)	2000 (1500-2800)	2000 (1400-2700)	0.018
Colloid (ml)	500 (500-1000)	750 (500-1000)	500 (500-1000)	0.003
RBC (U)	0 (0-0)	0 (0-2)	0 (0-0)	0.001
Plasma (ml)	0 (0-0)	0 (0-0)	0 (0-0)	0.001
Urine output (ml)	500 (280-700)	680 (400-1000)	600 (350-800)	<0.001
Furosemide (mg)	0 (0-0)	0 (0-0)	0 (0-0)	0.664
Estimated blood loss (ml)	100 (30-200)	150 (50-500)	100 (50-237)	0.003
Goal-directed fluid therapy	11 (9.5%)	14 (11.9%)	25 (10.7%)	0.555
Norepinephrine (mg)	0 (0-0.2)	0.6 (0.1-2.1)	0.2 (0-0.8)	<0.001

**Table 3:** Postoperative data between two groups.

Variables	Vasopressor use <3天 N=116	Vasopressor use≥3天 N=118	TOTAL N=234	P-value
ICU	27 (23.3%)	41 (34.7%)	68 (29.1%)	0.053
Crystal (ml/kg/d)	26.2 (18.8-38.8)	57.4 (43.8-70.8)	42.4 (25.5-60.2)	<0.001
RBC (U)	0 (0-0)	0 (0-0)	0 (0-0)	0.252
Plasma (ml)	0 (0-0)	0 (0-0)	0 (0-0)	0.189
24 h urine output (ml)	2150 (1500-2882)	2630 (2000-3400)	2400 (1752-3100)	<0.001
Furosemide (mg)	0 (0-0)	0 (0-20)	0 (0-0)	<0.001
Albumin (g)	0 (0-0)	0 (0-10)	0 (0-0)	<0.001
Hospital stay after Surgery(days)	7.0 (6.0-9.0)	10.0 (8.0-12.0)	8.0 (7.0-11.0)	<0.001

All variables with a P-value of <0.1 were included in the multivariate regression analysis (**Table 4**). The logistic regression analysis results indicated that the amount of preoperative colloid use (odds ratio [OR]=1.834, confidence interval [CI]:1.265–2.659, P=0.001), intraoperative booster use (OR=4.174, CI:1.882–9.258, P<0.001), and postoperative crystalloid solution input (ml/kg/d) (OR=1.087, CI:1.062–1.112, P<0.001) were risk factors for predicting postoperative hemodynamic instability (**Table 4**).

**Table 4:** Multivariable analysis for predictors of postoperative norepinephrine use reaching and exceeding the median number of days.

Variables	Odds ratio (95% CI)	P-value
preoperative colloid (ml)	1.834 (1.265-2.659)	0.001
Intraoperative vasopressor support	4.174 (1.882-9.258)	<0.001
postoperation crystal (ml/kg/d)	1.087 (1.062-1.112)	<0.001

CI, confidence interval.

**Figure 2:**
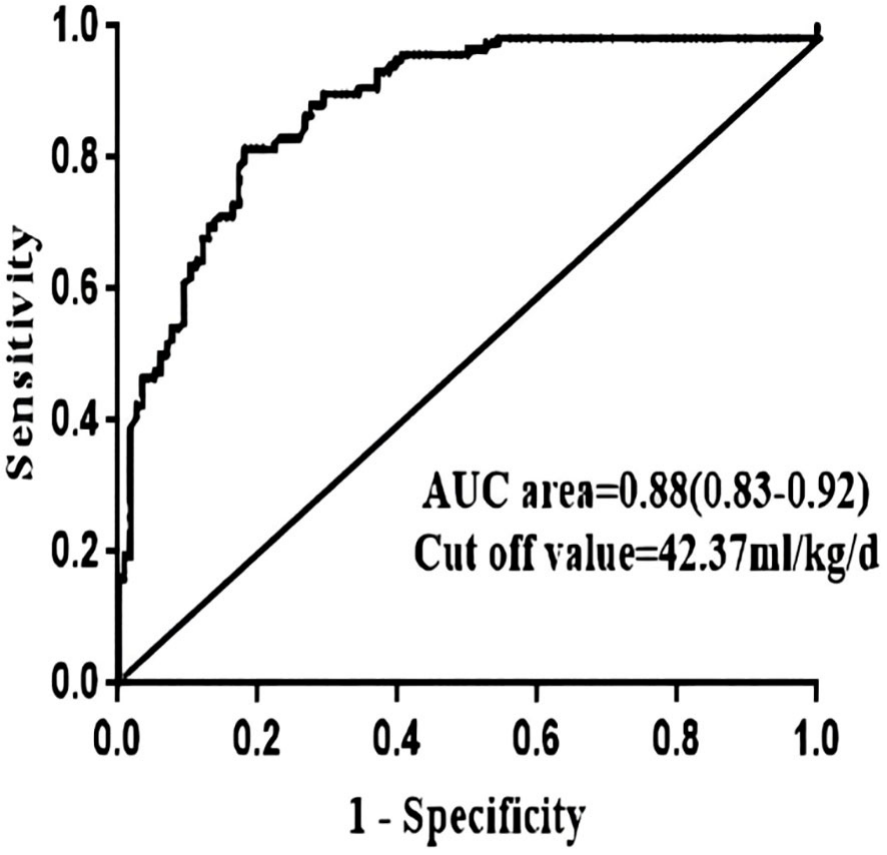
ROC curve of continuous independent risk factors (postoperative crystals) of norepinephrine use reaching and exceeding the median number of days. AUC, area under the curve.

In addition, the optimal cutoff point of the amount of postoperative crystalloid solution used were 42.37 ml/kg/d, which were analyzed using the receiver operating characteristic curve method (**Figure 2**).

## Discussion

There is no clear uniform definition of hemodynamic instability (12). In this study, the need for vasoactive drugs for a median number of days postoperatively was defined as hemodynamic instability in view of investigating the factors affecting hemodynamic stability following surgery.

Postoperative hemodynamic instability mainly refers to the postoperative development of hypotension, which requires the use of vasoactive drugs to maintain the blood pressure levels. According to current reports, the common causes of postoperative hypotension include a sudden drop in catecholamine levels following tumor resection or decreased vascular tone due to vascular insensitivity to catecholamines, decreased myocardial function that cannot compensate for peripheral vasodilation, relative fluid deficiency, residual effects of the preoperative use of alpha-adrenergic receptor blockers, or excessive intraoperative blood loss (8, 13). Various studies have suggested that the incidence of postoperative hypotension is approximately 30%–60%, which is consistent with the results of the present study (8, 14).

The results of the present study indicated that the intraoperative use of vasoactive drugs during pheochromocytoma resection is 3.174 times more likely to be a risk factor for the development of hemodynamic instability following surgery than the non-use of these drugs. In our study, intraoperative vasoactive drugs were used in 85.6% of the patients, and more than two-thirds of these patients had an intraoperative episode of hypotension requiring treatment. Previous studies have reported intraoperative hypotension requiring vasoactive drug as an independent risk factor for postoperative cardiovascular morbidity (15). Li et al. (16) also suggested that the presence of intraoperative hypotension is an independent risk factor for complications, while Pisarska-Adamczyk et al. (15) suggested that intraoperative treatment of hypotension with vasoactive drugs was the only risk factor for the development of postoperative hypotension. These findings are similar to those obtained in the present study.

The persistence of high levels of catecholamines in the body preoperatively may result in desensitization of the adrenergic receptors, which reduces the affinity of catecholamines for these receptors or decreases the number of cell surface receptors (17, 18). At this point, while the patient will likely have high levels of catecholamines in the body, they may not present the corresponding clinical manifestations and can be easily overlooked. Intraoperative ligation of tumor veins followed by catecholamine receptor desensitization or a dramatic decrease in catecholamines and the persistence of the effects of preoperative antihypertensive drugs may be possible causes of chronic vascular paralysis hypotension following a tumor resection requiring the use of vasoactive drugs to maintain the blood pressure (19).

Increased preoperative colloid input is also a predictor of the development of hemodynamic instability in the postoperative period, and this study’s findings revealed that for every 1,000-ml increase in the amount of colloid infused preoperatively, there is a 0.834-fold increase in the risk of developing hemodynamic instability in the postoperative period. It has been demonstrated that preoperative failure to enter crystalloid solution or colloids is an independent risk factor for postoperative cardiovascular morbidity (20). Recently, it has been recommended that all patients with a high preoperative suspicion of pheochromocytoma should be treated with appropriate infusion for volume expansion to avoid hemodynamic instability in the perioperative period (21, 22). However, there are differing views, with Hao et al. (23) suggesting that in patients undergoing pheochromocytoma resection, preoperative intravenous fluid replacement does not prevent perioperative hemodynamic changes, while Niederle et al. (24) performed goal-directed fluid therapy in patients undergoing pheochromocytoma resection using esophageal Doppler ultrasound to ascertain that the cause of postoperative hypotension was vascular paralysis, rather than true hypovolemia.

The results of this study indicated that the higher the postoperative crystalloid solution input, the higher the risk of postoperative hemodynamic instability. Thompson et al. (6) reported that the appearance of postoperative hypotension was transient and did not have serious consequences for postoperative outcomes, but was associated with increased intravenous input during the first 24 h postoperatively. The amount of crystal input in our study was calculated by comparing the amount per unit of body weight per day, and the results indicated that a higher amount of crystalloid solution input per unit of body weight per day postoperatively was associated with hemodynamic instability. On the one hand, due to the high function of the tumor, which causes vascular paralysis, more vasoactive drugs and fluid expansion are needed, while on the other, the patient’s surgical stress response and violent circulatory fluctuations may lead to intimal damage and capillary leakage, with <5% of the fluid volume remaining in the vessels after 1 h of infusion (25). Furthermore, the large amount of fluid input will aggravate any tissue interstitial edema. Fluid overload with excess volume may lead to decreased cardiac function (26, 27), increased intra-abdominal pressure (26–28), and increased renal venous congestion (29, 30). Elevated intra-abdominal pressure decreases renal perfusion (28) and venous return, with the decreased venous return leading to decreased cardiac output (31) and consequently exacerbating the onset of hypotension.

Fluid input and the use of vasoactive drugs are considered to be the main measures for treating postoperative hemodynamic instability in patients. A growing number of studies have proposed that preoperative volume expansion does not reduce the perioperative hemodynamic fluctuations or the occurrence of related complications, and does not improve the patient’s prognosis (32–35). This is largely because crystalloid fluid is free to cross the semi-permeable capillary intima and ends up being stored in the vasculature with only one-fifth of the input volume. While colloidal fluid has a stronger volume expansion effect, this effect only lasts for 24 h (36).

Meanwhile, the hazards associated with volume overload are gradually being appreciated. These hazards include slow repair of acute kidney injury, slow wound healing, prolonged mechanical ventilation (28, 37, 38), acute pulmonary edema, acute respiratory distress syndrome (38–43), and impaired cardiac function (44). Due to the low incidence of pheochromocytoma, most of the relevant studies are currently small-sample single-center retrospective studies. Among them, Niederle et al. (24) conducted a prospective study using minimally invasive hemodynamic monitoring for intraoperative goal-directed fluid therapy and found that patients do not benefit from the use of free fluid infusion and that perioperative volume overload should be avoided. However, the sample in this study was too small and this aspect must be further explored using larger-sample prospective studies.

## Conclusion and limitations

This study involved a number of limitations. First, while the factors affecting the intraoperative and postoperative hemodynamic stability of pheochromocytoma resection were comprehensively explored, the low incidence and the difficulty of preoperative diagnosis confirmation made it difficult to complete a randomized prospective study with a small sample size and limited reference space. Second, this study included patients who had a pathological indication of pheochromocytoma in our hospital over the last seven years, and both perioperative management and the surgical techniques have been constantly updated and the level of medical progress has been rapid, meaning the impact of these changes on the results is difficult to estimate. Third, urinary or plasma catecholamine monitoring was not performed, and its effect on hemodynamic instability could not be predicted. Fourth, since this was a retrospective study, all the data were obtained from electronic medical records, which may have resulted in a loss of accuracy and comprehensiveness of the assessment and documentation. While our study included cases of intraoperative goal-directed fluid therapy, it did not reveal a significant difference in terms of the improvement in postoperative hemodynamic instability. This may have been partly due to the limited number of cases and partly because our data were obtained from retrospective electronic medical records without a rigorous prospective study.

In conclusion, the instability and variability of perioperative blood flow in pheochromocytoma and the potential for dramatic intraoperative blood pressure fluctuations, even with adequate preoperative preparation, both surgery and anesthesia for pheochromocytoma should be performed by experienced surgeons who are constantly refining and updating their perioperative management strategies, further prospective randomized controlled studies must be conducted.

## Data availability statement

The raw data supporting the conclusions of this article will be made available by the authors, without undue reservation.

## Ethics statement

The studies involving humans were approved by Ethics Committee of Shengjing Hospital affiliated to China Medical University. The studies were conducted in accordance with the local legislation and institutional requirements. The participants provided their written informed consent to participate in this study. Written informed consent was obtained from the individual(s) for the publication of any potentially identifiable images or data included in this article.

## Author contributions

BL: Conceptualization, Writing – review & editing. LL: Data curation, Investigation, Methodology, Software, Supervision, Writing – original draft. LS: Data curation, Investigation, Methodology, Project administration, Software, Supervision, Validation, Writing – original draft. YZ: Formal analysis, Writing – review & editing. XS: Data curation, Writing – review & editing. XL: Data curation, Writing – review & editing. YS: Data curation, Writing – review & editing.
